# Evolutionary Analysis of DELLA-Associated Transcriptional Networks

**DOI:** 10.3389/fpls.2017.00626

**Published:** 2017-04-25

**Authors:** Asier Briones-Moreno, Jorge Hernández-García, Carlos Vargas-Chávez, Francisco J. Romero-Campero, José M. Romero, Federico Valverde, Miguel A. Blázquez

**Affiliations:** ^1^Instituto de Biología Molecular y Celular de Plantas, Consejo Superior de Investigaciones Científicas – Universidad Politécnica de ValenciaValencia, Spain; ^2^Institute for Integrative Systems Biology (I2SysBio), University of ValenciaValencia, Spain; ^3^Department of Computer Science and Artificial Intelligence, Universidad de SevillaSevilla, Spain; ^4^Instituto de Bioquímica Vegetal y Fotosíntesis, Consejo Superior de Investigaciones Científicas – Universidad de SevillaSevilla, Spain

**Keywords:** gene co-expression networks, integrative molecular systems biology, evo–devo, transcriptional regulation, plant signaling

## Abstract

DELLA proteins are transcriptional regulators present in all land plants which have been shown to modulate the activity of over 100 transcription factors in Arabidopsis, involved in multiple physiological and developmental processes. It has been proposed that DELLAs transduce environmental information to pre-wired transcriptional circuits because their stability is regulated by gibberellins (GAs), whose homeostasis largely depends on environmental signals. The ability of GAs to promote DELLA degradation coincides with the origin of vascular plants, but the presence of DELLAs in other land plants poses at least two questions: what regulatory properties have DELLAs provided to the behavior of transcriptional networks in land plants, and how has the recruitment of DELLAs by GA signaling affected this regulation. To address these issues, we have constructed gene co-expression networks of four different organisms within the green lineage with different properties regarding DELLAs: *Arabidopsis thaliana* and *Solanum lycopersicum* (both with GA-regulated DELLA proteins), *Physcomitrella patens* (with GA-independent DELLA proteins) and *Chlamydomonas reinhardtii* (a green alga without DELLA), and we have examined the relative evolution of the subnetworks containing the potential DELLA-dependent transcriptomes. Network analysis indicates a relative increase in parameters associated with the degree of interconnectivity in the DELLA-associated subnetworks of land plants, with a stronger effect in species with GA-regulated DELLA proteins. These results suggest that DELLAs may have played a role in the coordination of multiple transcriptional programs along evolution, and the function of DELLAs as regulatory ‘hubs’ became further consolidated after their recruitment by GA signaling in higher plants.

## Introduction

Higher plants are characterized by a particularly flexible capacity to adapt to multiple environmental conditions. In other words, environmental signals are very efficient modulators of plant developmental decisions. This ability is generally assumed to be based on at least two mechanistic features: the presence of an extensive and sensitive repertoire of elements that perceive environmental signals (such as light photoreceptors covering a wide range of wavelengths), and the high degree of interconnectivity between the different signaling pathways to allow cellular integration of variable information (Casal et al., 2004).

Evidence has accumulated in recent years about the important role that plant hormones play in the translation of environmental signals into developmental decisions. On one hand, it has become evident that hormone pathways share common components with the pathways that transduce light and other environmental signals (Jaillais and Chory, 2010); and, on the other hand, hormones have been shown to participate in the regulation of developmental processes all throughout a plant’s life cycle (Alabadi et al., 2009). In this context, gibberellins (GAs) and DELLA proteins are a paradigmatic example of the mechanisms that allow environmental signal integration. DELLA proteins constitute a small clade within the GRAS family of loosely defined plant specific nuclear proteins (Vera-Sirera et al., 2015). Their name was coined on the basis of a short stretch of amino acids (D-E-L-L-A) in their N-terminal region, which is tightly conserved among all higher plant species. They also present additional conserved motifs, such as the VHYNP domain, two leucine heptad repeats which may mediate protein–protein interactions, a putative nuclear localization signal, and a putative SH2 phosphotyrosine-binding domain, among others (Vera-Sirera et al., 2015). It has been shown in *Arabidopsis thaliana* and rice that recognition of GAs by their GID1 receptor allows physical interaction with DELLA proteins and promotes their degradation via the proteasome. In *A. thaliana*, loss of DELLA function mimics the phenotype of plants treated with an excess of GAs, both anatomically and also at the transcriptional level (Schwechheimer, 2011; Locascio et al., 2013b). Work in the past few years has established that DELLAs regulate transcription through the interaction with more than 100 transcription factors (TFs) in *A. thaliana* (de Lucas et al., 2008; Feng et al., 2008; Crocco et al., 2010; Hou et al., 2010; Gallego-Bartolomé et al., 2012; Daviere et al., 2014; Marin-de la Rosa et al., 2014, 2015; Resentini et al., 2015). In some cases, interaction with the TF inhibits its ability to bind DNA, while in other cases DELLAs seem to act as co-activators (Locascio et al., 2013b; Daviere and Achard, 2016). For all the cases examined in detail, the DELLA region responsible for the interaction with the TFs is the C-terminal region of the protein, the GRAS domain. Given that GA levels are strongly regulated by environmental signals such as light, temperature and photoperiod (Hedden and Thomas, 2012; Colebrook et al., 2014), cellular DELLA levels seem to be a proxy for the environmental status faced by plants (Claeys et al., 2014). Changes in DELLA levels could in turn differentially modulate distinct sets of TFs and their target genes in various developmental contexts. The promiscuous interaction with TFs, and the observation that *A. thaliana dellaKO* mutants display constitutive growth even under stress, and suffer from increased sensitivity to several types of stress factors such as salinity, cold, or fungal attacks (Alabadí et al., 2004; Achard et al., 2006, 2007, 2008a,b; Cheminant et al., 2011) suggests that DELLAs are potentially important ‘hubs’ in the transcriptional network that regulates the balance between growth and stress tolerance in higher plants.

Previous interest in the evolution of DELLA proteins is restricted to the question on how they were recruited to mediate cellular signaling by GAs. Based on phylogenetic analyses and shallow molecular analysis with fern and moss orthologs, it seems that the GA/GID1/DELLA module originated with early diverging tracheophytes (Wang and Deng, 2014). For instance, the *Selaginella* genus possesses the ability to synthesize GAs, a GID1 GA receptor, and a DELLA protein (Wang and Deng, 2014), which is sensitive to GA-induced degradation, even when introduced in an angiosperm, such as *A. thaliana* (Hirano et al., 2007; Yasumura et al., 2007). On the other hand, the DELLA proteins that existed in other land plants before the emergence of vascular plants were not involved in GA signaling. First, there are no *bona-fide DELLA* genes in algae and, second, the genomes of bryophytes like *Physcomitrella patens* encode DELLA proteins that lack the canonical ‘DELLA motif’ (Wang and Deng, 2014), and PpDELLAs are not sensitive to GAs when introduced in *A. thaliana* (Yasumura et al., 2007). However, the ability of DELLA proteins to modulate transcriptional programs relies on the GRAS domain through which interactions with TFs occur, and the evolution of this activity has not been addressed before.

In an attempt to identify the possible function of ancestral DELLAs and to delineate how evolution has shaped the functions of the GA/DELLA module in higher plants, we have addressed the analysis of the transcriptional networks potentially regulated by DELLAs in several species. For this reason, we have used gene co-expression networks, in which genes are represented as nodes, and if two genes exhibit a significant correlation value for co-expression, the corresponding nodes are joined by an edge. Importantly, if a node is a TF, first neighbors can be confidently taken as targets for that particular TF (Franco-Zorrilla et al., 2014). Therefore, the analysis of topological parameters of a gene co-expression network is an interesting tool that may reveal information about the function and evolutionary history of transcriptional programs (Aoki et al., 2007; Usadel et al., 2009).

**FIGURE 1:**
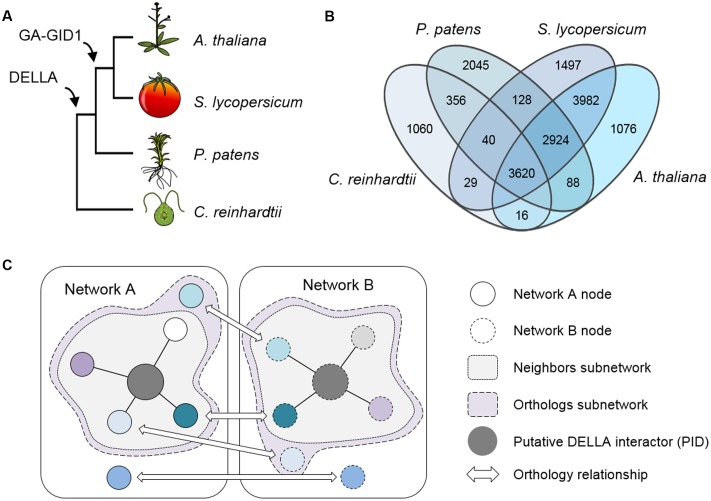
**Phylogenetic relationships between the chosen species. (A)** Representation of the species tree indicating the origin of key elements related to the gibberellin signaling pathway. **(B)** Venn’s diagram showing the number of OrthoMCL groups in which genes of each species are present. **(C)** Schematic representation of the basis for subnetwork design. Nodes in different networks with the same color indicate an orthologous relationship.

Here we have investigated the properties of networks formed by DELLA-interacting TFs and their co-expressing genes in *A. thaliana*, and compared them with the orthologous networks in three other plant species: (i) *Solanum lycopersicum* (possessing a fully operative GA/DELLA module); (ii) *P. patens* (possessing GA-independent DELLA functions); and (iii) *Chlamydomonas reinhardtii* (without GA perception or DELLAs) (**Figure 1A**). All the parameters examined suggest that the functions regulated by DELLA-interacting TFs (and thus DELLAs themselves) have increased their level of coordination along evolution.

## Results and Discussion

### Construction of Networks and Subnetworks

Gene expression data from RNA sequencing (RNA-seq) experiments in *A. thaliana*, *S. lycopersicum*, *P. patens*, and *C. reinhardtii* were obtained from the Gene Expression Omnibus, and gene co-expression networks were inferred for each species from transcriptomic data as described in section “Materials and Methods.” All four networks are scale-free networks (Supplementary Figure S1) (Romero-Campero et al., 2013, 2016) and have comparable sizes in terms of number of nodes, but there are remarkable differences in the way they are connected (**Table 1**). The *A. thaliana* network contains more than twice as many edges than the others, the average degree of its nodes (average number of connections) is one order of magnitude higher and its average shortest path length (average number of nodes between two random nodes) is lower. Even though the number of genes of each species represented in the networks is similar, in some species they are more connected, possibly due to differences in their endogenous regulation and the availability of experimental data. For that reason, we decided to do every comparative analysis between the different species in relative terms.

**Table 1:** General parameters in co-expression networks.

	*C. reinhardtii*			*P. patens*			*S. lycopersicum*			*A. thaliana*		
	Full	Neigh	Ortho	Full	Neigh	Ortho	Full	Neigh	Ortho	Full	Neigh	Ortho
Nodes	8652	48	658	8564	448	1503	7851	1314	2885	5663	2070	2949
Edges	145903	78	1173	295317	15078	19828	287409	153396	169171	593730	460951	512042
Average degree	33.73	3.25	3.57	68.97	67.31	26.38	73.22	233.48	117.28	209.69	445.36	347.26
Average shortest path length	7.37	1.91	8.71	13.11	1.39	12.01	13.78	1.67	5.63	4.28	2.15	3.09
Diameter	23	4	24	46	4	41	44	6	25	20	9	12

*Parameters of networks and subnetworks used in this study. Full, full gene co-expression network; Neigh, first neighbors subnetwork; Ortho, orthologs subnetwork; *C. reinhardtii*, *Chlamydomonas reinhardtii*; *P. patens*, *Physcomitrella patens*; *S. lycopersicum*, *Solanum lycopersicum*; *A. thaliana*, Arabidopsis thaliana*.

To be able to compare the co-expression networks of the different species, we first identified the orthologous nodes in each of them using the OrthoMCL method (Li et al., 2003). Up to 17,053 groups of genes were obtained. Genes in the same group were considered orthologs or paralogs if they belonged to different or the same species, respectively. The four species were represented unequally, as both *A. thaliana* and *S. lycopersicum* genes were present in ca. 70% of the groups, while *P. patens* genes were found in little more than 50% of them, and only ca. 30% of the groups contained genes from *C. reinhardtii* (**Figure 1B**). This was already expected, given the evolutionary distance among these species and the genomic complexity of each one.

**FIGURE 2:**
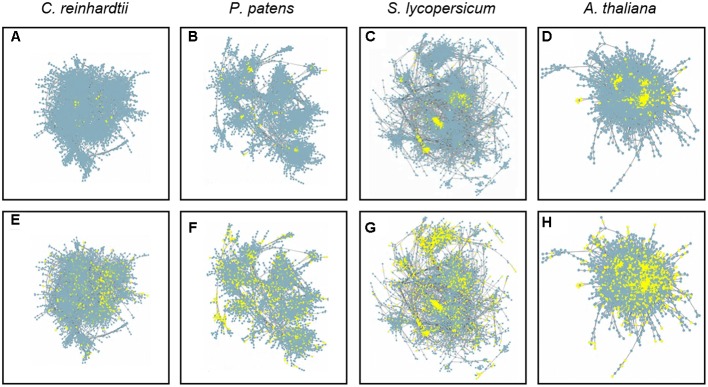
**Gene co-expression networks.** Full *Chlamydomonas reinhardtii* **(A,E)**, *Physcomitrella patens* **(B,F)**, *Solanum lycopersicum* **(C,G)**, and *Arabidopsis thaliana* **(D,H)** gene co-expression networks. Neighbors subnetworks are comprised of yellow-marked nodes in A-D. Orthologs subnetworks are comprised of yellow-marked nodes in **(E–H)**.

To assess the possible contribution of DELLA proteins to co-expression networks architecture, we created subnetworks based on reported DELLA interactors known to act as transcriptional regulators. First, we compiled a list of all published DELLA interactors (**Supplementary Table S1**), obtained their orthologs for the four species, and localized them in their respective networks. Since most of the interactions have been described for *A. thaliana*, the corresponding orthologs in the other species are only “putative interactors of the DELLA proteins” (PIDs), and the first neighbors of AtDELLA interactors and PIDs are their putative expression targets. Second, we built two different subnetworks using this information. The first one, called “Neighbors” subnetwork (abbreviated as AtNeigh, SlNeigh, PpNeigh, and CrNeigh), is composed of the DELLA interactors (or the corresponding PIDs) and their first neighbors (**Figure 1C** and **Supplementary Table S2**). The second one, called “Orthologs” subnetwork (abbreviated as AtOrtho, SlOrtho, PpOrtho, and CrOrtho), contains the orthologs of all the first neighbors of PIDs in all the species (**Figure 1C** and **Supplementary Table S3**). For a given species, the “Neighbors” subnetwork provides a good approximation to its actual DELLA-dependent transcriptome, while the “Orthologs” subnetwork represents the full landscape of potential transcriptional targets for DELLAs, since it includes orthologs of genes that are DELLA transcriptional targets in other species (**Figure 2**).

### DELLA-Associated Subnetworks Reflect Increased Relevance of DELLAs after Being Recruited by GA Signaling

It is important to take into account a circumstance that affects the construction of subnetworks: OrthoMCL does not always retrieve orthologs for some of the genes, because either they do not exist in the other species, or the method does not provide high-confidence results. This results in a particular bias toward smaller subnetwork sizes with increasing phylogenetic distance (**Table 1**). However, the impact of this bias can be disregarded when analyzing relative parameters. Hence, regardless of the absolute sizes, we observed that the average degree in the Neighbor subnetworks increased dramatically in SlNeigh and AtNeigh with respect to their full networks (more than threefold and twofold, respectively), while this parameter did not change in PpNeigh, and it actually decreased in CrNeigh (**Table 1**). Similarly, the Orthologs subnetworks displayed an equivalent behavior as the Neighbors subnetworks: their diameter and average shortest path length decreased considerably more in SlOrtho and AtOrtho with respect to the full networks; and the same happened with the increase of the average degree. In summary, both subnetworks showed a higher compaction and interconnection of nodes in relative terms in the case of *S. lycopersicum* and *A. thaliana* compared with *P. patens* and *C. reinhardtii*, indicating that the putative interactors and targets of the DELLAs become more connected in those species presenting GA-regulated DELLAs.

**FIGURE 3:**
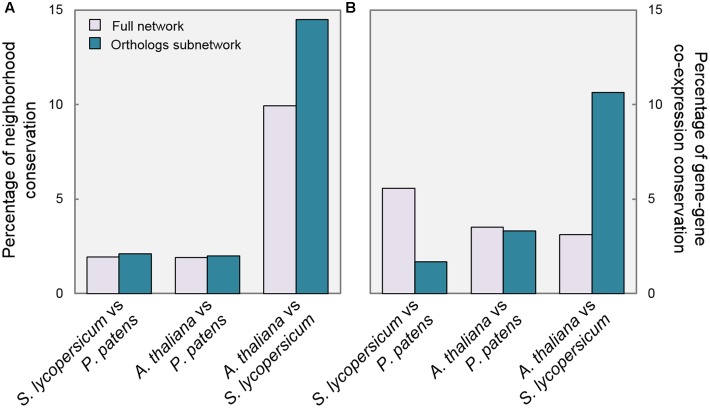
**Gene connections are more conserved in species with GA-regulated DELLAs.** Pairwise comparisons of *P. patens*, *S. lycopersicum*, and *A. thaliana* Full networks and Ortho subnetworks regarding: **(A)** Percentage of genes with significantly overlapping neighborhoods; **(B)** Percentage of conserved gene–gene links.

A confirmation of the impact of GA regulation on the relevance of DELLA function is found in the analysis of neighborhood conservation. **Figure 3A** shows the percentage of genes with a significantly overlapping neighborhood in each comparison (see Materials and Methods). When comparing *P. patens* with the other species, there are no substantial differences between the full network and the Orthologs subnetwork. On the contrary, SlOrtho and AtOrtho contain a considerably higher proportion of genes with conserved neighborhood than their corresponding full networks (15% vs. 10%). Between *S. lycopersicum* and *A. thaliana*, the regulation of the putative DELLA targets is more conserved than for the network in general, so this group of genes seems to have a cohesive element in the two species.

Furthermore, we examined gene–gene co-expression values, as a measure of the conservation of individual edges. For every pair of linked genes in one species, if the corresponding orthologs are also linked in a second species, it is considered that gene–gene co-expression is conserved. Therefore, the calculation of conserved links between two subnetworks is a measure of functional conservation of a regulatory module. Interestingly, we observed that gene links between PpOrtho and SlOrtho were less conserved than in the full networks, and almost unaltered between PpOrtho and AtOrtho (**Figure 3B**). However, the gene–gene co-expression was three times more conserved between SlOrtho and AtOrtho than between their full networks (11% vs. 3.5%). In other words, these data are compatible with the proposition that the presence of GA-regulated DELLAs (in *S. lycopersicum* and *A. thaliana*) provides stronger links between transcriptional programs, not detected in an organism with GA-independent DELLAs (*P. patens*).

### Efficiency of Transcriptional Regulation Is a DELLA-Associated Parameter

**FIGURE 4:**
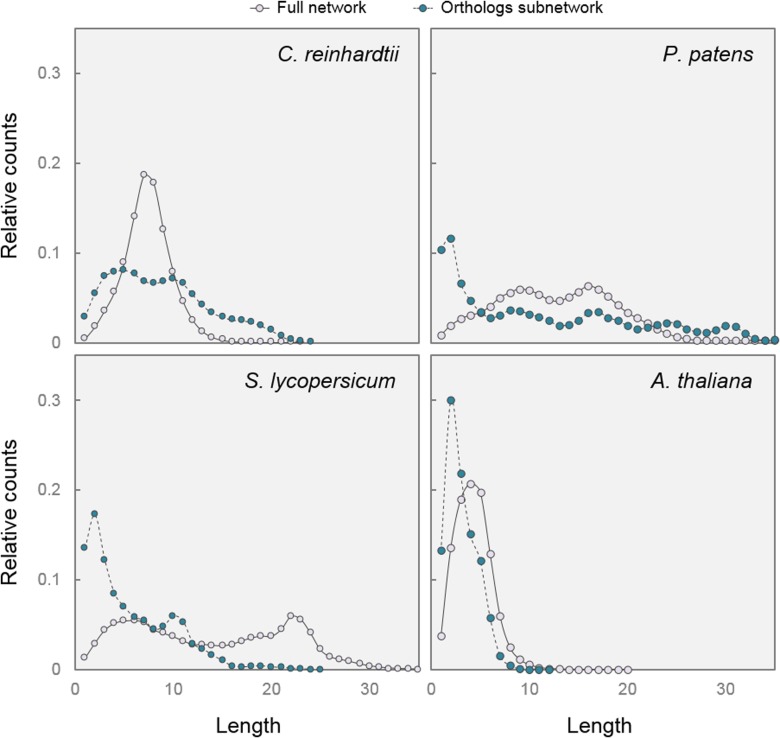
**Paths are shorter in DELLA-associated subnetworks.** Shortest path length distribution in Full networks and Orthologs subnetworks from the four species. The graphs represent the relative number of nodes (*y*-axis) joined by a given number of intermediate nodes (*x*-axis).

The efficiency of a transcriptional regulatory mechanism can be evaluated through two additional parameters in gene co-expression networks: shortest path length distribution and motif frequency. In network theory, average shortest-path length is defined as the average number of steps along the shortest paths for all possible pairs of network nodes. It is a measure of the efficiency of information propagation on a network, with a shorter average path length being more efficient (Vragovic et al., 2005). When we compared the distribution of shortest path lengths in full and Orthologs subnetworks, we observed a clear tendency toward shorter path lengths in the Orthologs subnetworks of organisms possessing DELLAs (*S. lycopersicum*, *A. thaliana*, and *P. patens*) compared with the situation in an organism without DELLAs (*C. reinhardtii*) (**Figure 4**).

**FIGURE 5:**
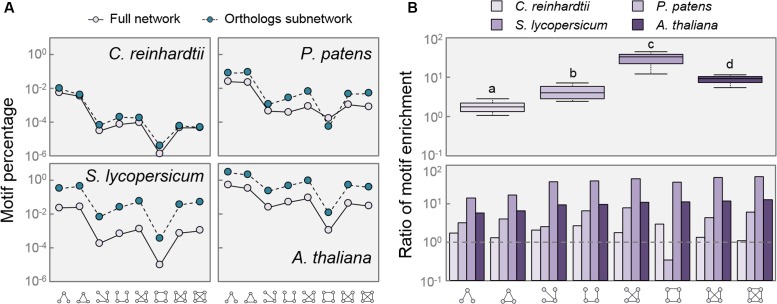
**Network motifs are enriched in DELLA related networks. (A)** Percentage of motifs found in each network compared to possible combinations of three and four nodes. **(B)** Ratio of motif enrichment comparing Orthologs subnetworks to Full networks per species (upper panel), and per motif (lower panel). Dashed lines in **(B)** mark a ratio of 1. Motifs are as depicted in *X*-axis. Letters indicate significant differences between groups, *p* < 0.01 (One way ANOVA, Tukey HSD *Post Hoc* test). Box-plot whiskers are Tukey-defined (extended 1.5 times the IQR from the box edges).

Network motifs are small recurring patterns involving a few nodes that appear more frequently in biological networks than in randomized ones. They consist of a certain level of regulation which connects small sets of nodes with a particular topology. Motifs characterize a network, as some of them are useful for the regulation of determined functions, and thus conserved along evolution (Kashtan and Alon, 2005). After measuring the frequency of the eight common motifs composed of three and four nodes in the full networks, we found that there was no relative enrichment of any particular motif between species when comparing the full networks or the Orthologs subnetworks (**Figure 5A**). However, the AtOrtho, SlOrtho, and PpOrtho subnetworks displayed a clear enrichment in virtually every motif, compared with their respective full networks (**Figure 5B**). Given that the function of this sort of motifs is to allow coordinated expression of a group of genes with shared function (Alon, 2007), the increase in the proportion of small regulatory patterns among all the putative DELLA targets in species that do contain DELLAs indicates an increase in the complexity of gene regulation, in which DELLAs might mediate the coordination of transcriptional programs.

### The Regulation of the Stress Response: A Likely Role of Ancestral DELLA Proteins

The results shown above suggest that the origin of DELLAs in land plants would be associated to an increase in the co-expression between genes that are putative targets of DELLA-interacting TFs, both in terms of size of the gene set and degree of the co-expression value. Therefore, DELLAs would have helped in the coordination of certain transcriptional circuits, and their recruitment to mediate GA signaling later in development would have further expanded their coordination capacity. To reveal the most likely functions ultimately regulated by DELLAs in the common ancestor of land plants, we carried out Gene Ontology (GO) analyses on each of the Neighbor subnetworks, with the idea that the terms shared by those in *S. lycopersicum*, *A. thaliana*, and *P. patens* could represent likely functions regulated by the ancestral DELLA proteins.

**FIGURE 6:**
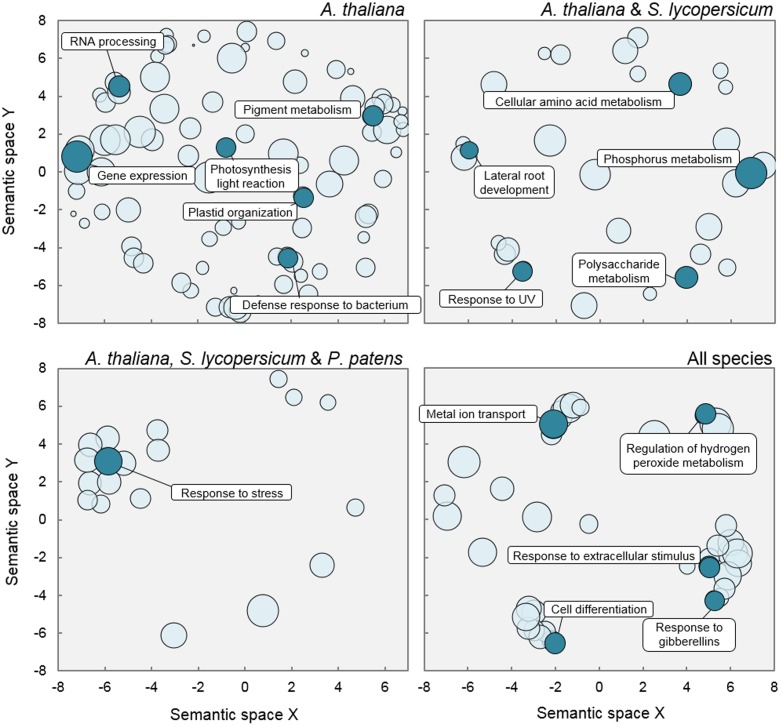
**Gene Ontology terms enriched in Neighbors subnetworks.** Scatterplots show cluster representatives after redundancy reduction in a two dimensional space derived by applying multidimensional scaling to a matrix of the GO categories semantic similarities. Bubble size is proportional to *p*-value significance of GO enrichment.

Not surprisingly, given the larger size of AtNeigh (**Table 1**), GO analysis rendered a much larger number of terms significantly enriched in this subnetwork, compared to those from the other three organisms (**Supplementary Table S4**). Terms referring to chloroplast function, such as plastid organization, photosynthesis, or pigment biosynthesis (including chlorophyll) were specifically enriched among the putative DELLA targets in *A. thaliana* only (**Figure 6**). This result might reflect functions whose regulation by DELLA has been acquired more recently, or it could simply be a bias of the analysis, caused by the big difference in size of the analyzed sets in the different species. On the contrary, the finding that terms comprised under general ‘response to stress’ were significantly over-represented in the subnetworks of the three land plants, but not *C. reinhardtii*, suggests that this function might have been the primary target of the regulation by ancestral DELLAs through their interaction with specific TFs.

## Conclusion

Our analysis suggests that DELLAs may have contributed to the acquisition of an increasing degree of coordination between transcriptional programs during plant evolution. Although these results are consistent with the current view of DELLAs as ‘hubs’ in transcriptional programs in higher plants, and provide a plausible evolutionary scenario, it is important to remark that further experimental work is required to validate most of the conclusions from *in silico* network analysis. In fact, several reasonable assumptions have been made that would be relatively easy to confirm. For instance, actual transcriptomic data of *dellaKO* mutants in the different species, coupled to comparative analysis would help establish the role of ancestral DELLAs. Moreover, our current analysis would be strengthened by the experimentally obtained information of which PIDs are in fact *bona-fide* DELLA interactors in the different species. Finally, the conclusion that DELLAs have probably contributed to the establishment of new co-regulatory circuits during land-plant evolution does not explain the molecular mechanism that supports this progressive acquisition, and it can be generated by changes in DELLA proteins, in their interactors, or in both.

## Materials and Methods

### Gene Co-expression Network Inference

The *C. reinhardtii* and *A. thaliana* networks were downloaded from the web resources of previous work (Romero-Campero et al., 2013, 2016). For the new networks, RNA-seq data were selected from equivalent experiments involving comparable tissues and environmental situations (**Supplementary Table S5**). The *P. patens* gene co-expression network was inferred from the RNA-seq data freely available from the Gene Expression Omnibus identified with accession numbers GSE19824, GSE33279, GSE36274, and GSE25237. The *S. lycopersicum* network was constructed based on the RNA-seq data identified with the accession numbers GSE45774, GSE64665, GSE64981, GSE68018, and GSE77340 in the Gene Expression Omnibus. In both cases, RNA-seq data was processed using the Tuxedo protocol (Trapnell et al., 2012) to obtain gene expression levels measured as FPKM. Briefly, short reads were mapped to the corresponding reference genome using Tophat, transcripts were assembled using Cufflinks and expression levels were computed using Cuffdiff. The Bioconductor R package cummeRbund (Goff et al., 2013) was used for subsequent analysis of the results generated by the Tuxedo protocol. In order to reduce noise in our analysis only genes that were detected as differentially expressed in at least one of the studies integrated in this work were considered. Differentially expressed genes were determined comparing each condition with the corresponding control within each study using a fold-change threshold of two. For each species, a matrix containing the expression levels of the selected genes was extracted. The Pearson correlation coefficient between every pair of gene expression profiles was computed using the *cor* function from the stats R package to generate a correlation matrix. Two genes were assumed to be co-expressed when the Pearson correlation coefficient between their expression profiles over the analyzed conditions was greater than 0.95. Following this criterion, the corresponding adjacency matrix was generated from the correlation matrix. Using the R package igraph^1^ (Csardi and Nepusz, 2006), each network was constructed from its adjacency matrix and exported in gml formal for subsequent analysis.

### Data Compilation and Processing

The reference proteomes from *A. thaliana* TAIR10, *S. lycopersicum* iTAGv2.3, *C. reinhardtii* v5.5, and *P. patens* v3.3 were downloaded from Phytozome (Goodstein et al., 2012). From all the possible proteins from each locus tag only the longest protein was kept and assigned to its locus tag. These files were used to identify the orthologs among the four species with OrthoMCL (Li et al., 2003).

The networks were converted to SIF format and processed using the package igraph^1^ (Csardi and Nepusz, 2006) made with R^2^ (R Core Team, 2016). Only the edges between two non-identical nodes were conserved. If a given node was not identified in the proteome files, it was removed from the network. Afterward, components with fewer than seven elements were removed from the network to generate the complete network for each species. The orthologs for the set of manually curated DELLA interactors from *A. thaliana* were identified, and these nodes were selected from the complete networks. The first neighbors for all the selected nodes were identified and used to build a subnetwork. Finally, the orthologs on each species for all the genes in the previous subnetworks were identified and used to generate a new subnetwork for each species.

### Network Analysis and Visualization

All networks were imported into the software package Cytoscape (Smoot et al., 2011) for their visualization using the Prefuse Force Directed layout.

The measures of network topology were calculated using both predefined and custom made functions. The gene–gene co-expression and neighborhood conservation were determined following the approach described by Netotea et al. (2014), using Fisher exact tests to check for statistical significance.

Gene Ontology analysis on Neigh subnetworks was made with AgriGO (Du et al., 2010), and represented with ReviGO (Supek et al., 2011).

## Author Contributions

AB-M, JH-G and MB conceived and designed the work. FR-C, JR, and FV constructed the co-expression networks. AB-M, CV-C, and JH-G performed network analyses. AB-M and MB wrote the first draft of the manuscript, to which all authors contributed.

## Conflict of Interest Statement

The authors declare that the research was conducted in the absence of any commercial or financial relationships that could be construed as a potential conflict of interest.

## Footnotes

1 http://igraph.com

2 http://www.Rproject.org

## Supplementary Material

TABLE S1 **Compilation of DELLA interactors used in this study**.

TABLE S2 **Genes included in the ‘Neighbors’ subnetworks**.

TABLE S3 **Genes included in the ‘Orthologs’ subnetworks**.

TABLE S4 **Gene Onthology categories enriched in the ‘Neighbors’ subnetworks**.

TABLE S4 **Gene Onthology categories enriched in the ‘Neighbors’ subnetworks**.
