# Supplementary material for: Evolutionary Analysis of DELLA-Associated Transcriptional Networks
Source: Front Plant Sci. 2017 Apr 25;8:626. doi: 10.3389/fpls.2017.00626 (PMC5404181; doi:10.3389/fpls.2017.00626)
Supplement: TABLE S4 — Gene Onthology categories enriched in the ‘Neighbors’ subnetworks. [file Table_5.DOCX]

| ***Physcomitrella patens*** | | |
| --- | --- | --- |
| **Study** | **GEO Accession** | **Overall Experimental Design** |
| Genome-wide transcriptome analysis of gametophyte development in *Physcomitrella patens* | GSE33279 | Samples were taken from protonema at 3, 14 and 24 days, from leafy shoot tissues at 30 days after protoplast isolation, and from 14-day-old caulonemal and chloronemal tissues. |
| Distinct phytochrome actions in nonvascular plants revealed by targeted inactivation of phytobilin biosynthesis | GSE36274 | Samples were taken from wild type and *pubs/hy2* double mutant protonema grown in the dark and exposed to red light for one hour. |
| The DNA damage response transcriptome of the moss *Physcomitrella patens* | GSE25237 | Samples were taken from untreated wild type tissue and tissue treated with the DNA-DSB inducing agent, bleomycin. |
| Genome-wide analysis of *Physcomitrella patens* DNA methylation and gene expression | GSE19824 | Samples consist of whole plants. |
| ***Solanum lycopersicum*** | | |
| **Study** | **GEO Accession** | **Overall Experimental Design** |
| Comparative transcriptomics reveals patterns of selection in domesticated and wild tomato | GSE45774 | Samples were taken from whole seedlings, inflorescences, leaves, roots and stems, vegetative meristems under artificial sun and shade. |
| Gene Expression Programs in Developing Roots from *Solanum lycopersicum* | GSE64665 | Samples were taken from developing roots 6 days after plating from meristematic, elongation and differentiation zones. |
| Transcriptome Profiling of Tomato Fruit Development | GSE64981 | Samples were taken from developing fruits at 7, 14, 21, 28, 35, 42 and 49 days after flowering. |
| Uncovering DELLA Independent Gibberellin Responses by Characterizing New Tomato procera Mutants. | GSE68018 | Samples were taken from GA untreated and treated wild type and *pro∆GRAS* seedlings young leaves. |
| Overexpression of SlRBZ results in etiolation and dwarfism in *Solanum lycopersicum* | GSE77340 | Samples were taken from young primary leaves from wild type and *SlRBZ* overexpressing transgenic plants. |
