# Supplementary material for: Evolutionary Analysis of DELLA-Associated Transcriptional Networks
Source: Front Plant Sci. 2017 Apr 25;8:626. doi: 10.3389/fpls.2017.00626 (PMC5404181; doi:10.3389/fpls.2017.00626)
Supplement: Supplementary file 6 [file Image_1.PDF]

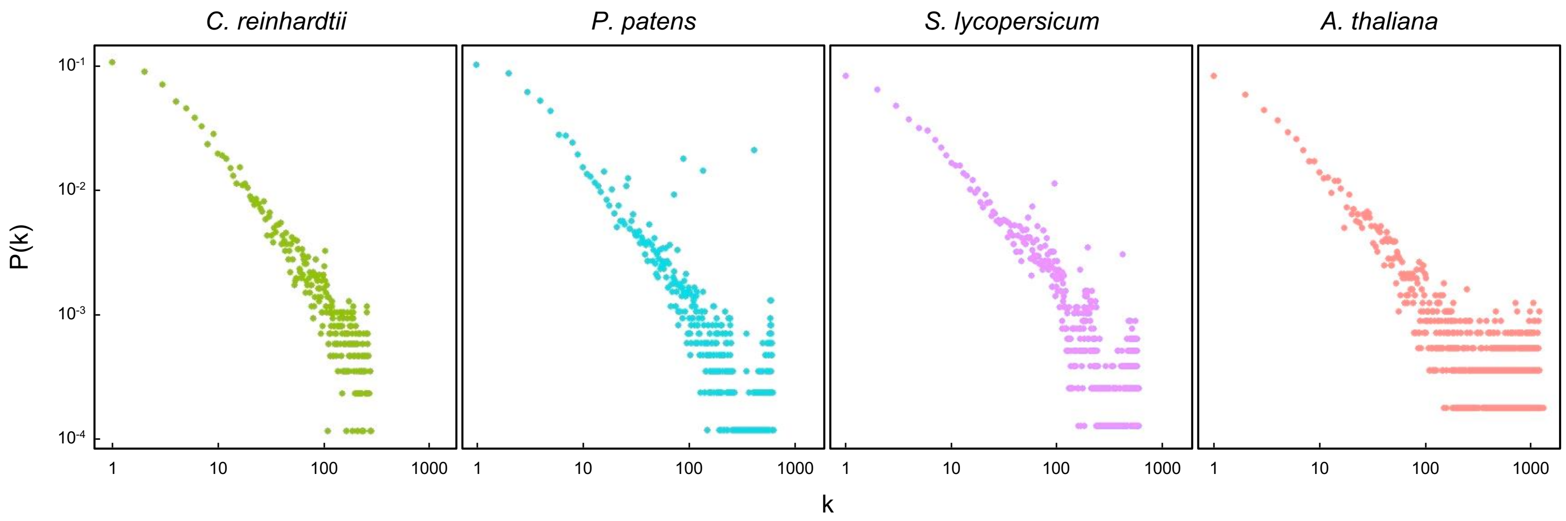

Supplementary Figure 1. Degree distribution of the gene co-expression networks constructed for each of the four species. P(k) is the proportion of nodes with k edges, represented in logarithmic scale. Biological networks like the ones presented here are usually scale-free (most nodes have few edges, while a small set of nodes have multiple connections).
